# The key role of the ferroptosis mechanism in neurological diseases and prospects for targeted therapy

**DOI:** 10.3389/fnins.2025.1591417

**Published:** 2025-05-12

**Authors:** Chenyu Xie, Nan Wu, Jiaojiao Guo, Liangliang Ma, Congcong Zhang

**Affiliations:** ^1^Department of Rehabilitation, The First Affiliated Hospital of Henan University of Chinese Medicine, Zhengzhou, China; ^2^Rehabilitation Medicine College, Henan University of Chinese Medicine, Zhengzhou, Henan, China

**Keywords:** ferroptosis, neurological diseases, cell death, research progress, medication therapy

## Abstract

Neurological disorders represent a major global health concern owing to their intricate pathological processes. Ferroptosis, defined as a form of cell death that is reliant on iron, has been closely linked to various neurological conditions. The fundamental process underlying ferroptosis is defined by the excessive buildup of iron ions, which initiates lipid peroxidation processes leading to cellular demise. Neurons, as highly metabolically active cells, are susceptible to oxidative stress, and imbalances in iron metabolism can directly initiate the ferroptosis process. In neurodegenerative disorders like Alzheimer’s disease and Parkinson’s disease, ferroptosis driven by iron accumulation represents a fundamental pathological connection. Although the connection between ferroptosis and neurological diseases is clear, clinical application still faces challenges, such as precise regulation of iron metabolism, development of specific drugs, and assessment of efficacy. The limited comprehension of the ferroptosis mechanism hinders the development of personalized treatment approaches. Consequently, subsequent investigations must tackle these obstacles to facilitate the clinical application of ferroptosis-associated therapies in neurological disorders. This article provides a comprehensive overview of the most recent advancements regarding the underlying mechanisms of ferroptosis. Subsequently, the study investigates the mechanistic contributions of ferroptosis within the nervous system. In conclusion, we evaluate and deliberate on targeted therapeutic strategies associated with ferroptosis and neurological disorders.

## 1 Introduction

Neurological disorders pose a significant and growing global health burden, with incidence rates increasing by 38% since 2000 (GBD, 2024). The World Health Organization reports that neurodegenerative diseases now affect 10.3% of adults aged ≥65 years, with Alzheimer’s disease (AD) incidence reaching 8.1 per 1,000 person-years and Parkinson’s disease (PD) at 3.4 per 1,000 person-years—representing 27 and 18% increases, respectively, since 2015 (Farzan et al., 2024; Alkhazaali-Ali et al., 2024). Emerging post-pandemic data further highlight the escalating neurological burden, with significantly elevated HRs (>1) for ischemic stroke and cognitive and memory disorders. Additionally, during recovery, COVID-19 survivors face an 80% higher risk of epilepsy (HR = 1.80, 95% CI 1.61–2.01) and a 50% increased risk of Parkinson’s disease (PD, HR = 1.50, 95% CI 1.28–1.75; Spudich and Nath, 2022). Furthermore, the prevalence of stroke, epilepsy, and multiple sclerosis has shown variable but consistently rising trends across different global regions (Hill and Peate, 2023). These disorders severely impair patients’ quality of life and impose substantial economic costs on healthcare systems worldwide.

Brent R. first proposed ferroptosis in 2012 as a form of programmed iron-dependent cell death, exhibiting unique characteristics at morphological, chemical, and genetic levels compared to other forms of cell death, such as apoptosis, necrosis, and autophagy (Li and Huang, 2022). Mechanistic studies reveal that neuronal ferroptosis susceptibility stems from three synergistic factors: high baseline iron retention in substantia nigra and hippocampal regions, GPX4 expression levels 40% lower than in glial cells, and preferential accumulation of polyunsaturated fatty acids in neuronal membranes (Sharma and Flora, 2021). These molecular vulnerabilities have spurred development of targeted therapeutics, with iron chelators (deferiprone), GPX4 stabilizers (Liproxstatin-1), and lipid antioxidant nanoparticles demonstrating 30–50% neuroprotection in Parkinson’s and Alzheimer’s models by specifically counteracting the three susceptibility factors. These factors underscore ferroptosis’s key role in neuronal vulnerability and disease mechanisms.

While the association between ferroptosis and neurological disorders has been increasingly elucidated, current research predominantly focuses on individual diseases or isolated mechanisms, lacking integrative analyses of ferroptosis-related common pathogenic mechanisms across different neurological disorders. This article addresses critical gaps in ferroptosis research by: (1) Systematically analyzing shared mechanisms across neurodegenerative diseases, acute brain injury, and neuroimmune disorders, revealing ferroptosis as a molecular bridge linking diverse pathologies; (2) Establishing a multi-target therapeutic framework integrating nanodelivery systems; (3) Pioneering spatiotemporal mapping of ferroptosis heterogeneity through single-cell sequencing-metabolomics integration to guide personalized interventions. These innovations provide transformative insights for developing broad-spectrum neuroprotective therapies and accelerating clinical translation.

## 2 Core mechanisms of ferroptosis

**Figure 1:**
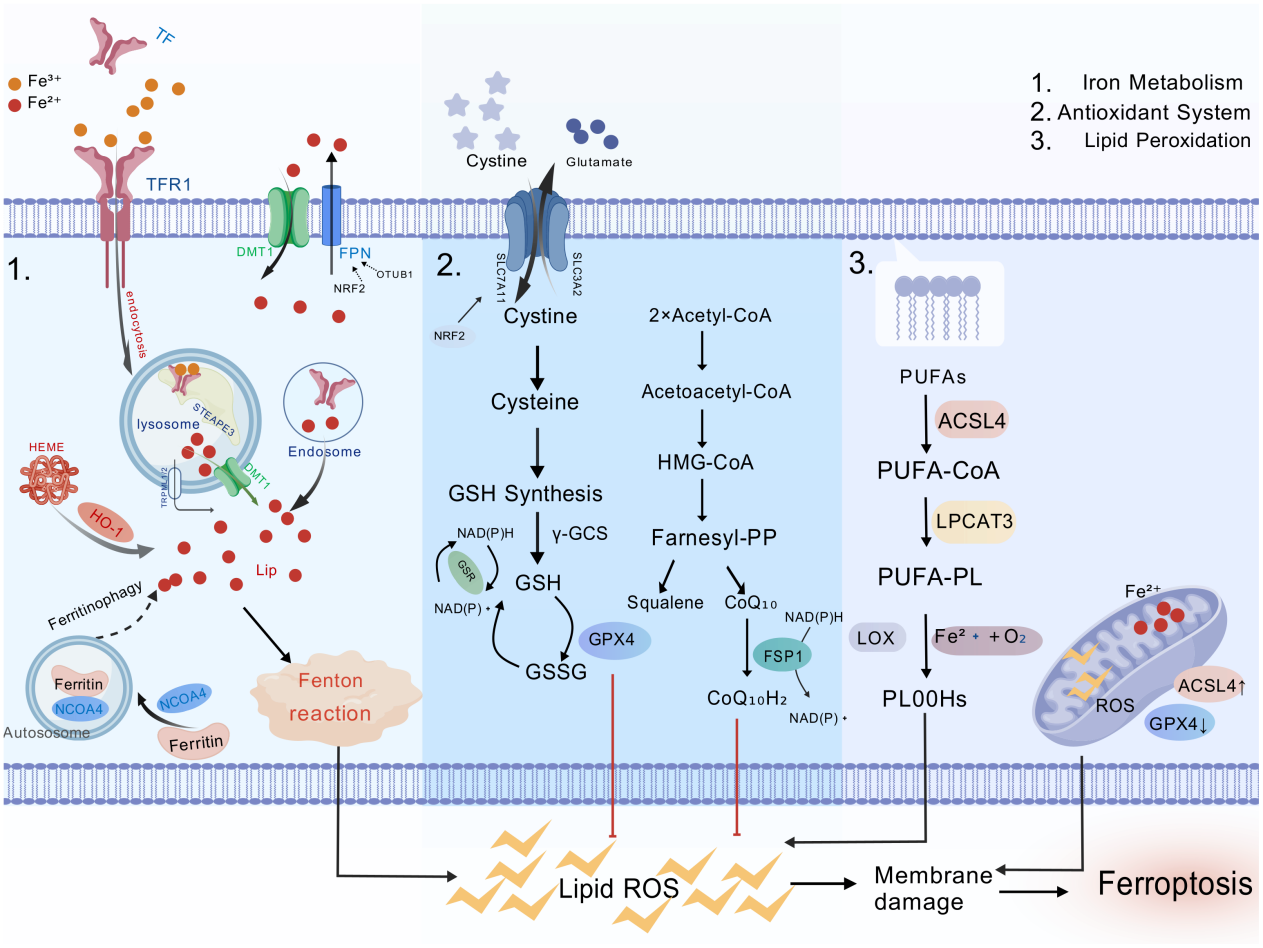
Created with Biogdp.com (Jiang et al., 2025). Tf, Transferrin; Fr1, Transferrin Receptor 1; Dmt1, Divalent Metal Transporter 1; Fpn, Ferroportin; Nrf2, Nuclear Factor Erythroid 2-Related Factor 2; Otub1, Otubain 1;Slc7A11, Solute Carrier Family 7 Member 11; Slc3A2, Solute Carrier Family 3 Member 2;Ho-1, Heme Oxygenase-1; Ncoa4, Nuclear Receptor Co-Activator 4; Steap3, Six-Transmembrane Epithelial Antigen of Prostate 3; Lip, Lipid; *γ* – Gcs, γ-Glutamyl Cysteine Synthetase; Gssg, Oxidized Glutathione; Acsl4, Acyl-CoA Synthetase Long-Chain Family Member 4; Lpcat3, Lysophosphatidylcholine Acyltransferase 3; Lox, Lipoxygenase; Pufas, Polyunsaturated Fatty Acids; Pufa-CoA, Polyunsaturated Fatty Acyl-Coenzyme A; Pufa-Pl, Polyunsaturated Fatty Acid-Phospholipid; Pl-Oohs, Phospholipid Hydroperoxides; CoQ₁₀, Coenzyme Q₁₀; Fsp1, Ferropoptosis Suppressor Protein 1. This is a diagram of the ferroptosis mechanism. On the left side, iron metabolism is shown. The middle part involves the antioxidant system. The right side demonstrates that lipid peroxidation is related to ferroptosis. Overall, it elucidates the interactions among various metabolic pathways and associated molecules involved in the process of ferroptosis.

As shown in Table 1, this table comprehensively summarizes the key aspects of each sub-topic, such as iron metabolism imbalance, lipid peroxidation, antioxidant system failure, and mitochondrial dysfunction under this main title, providing a foundation for the following in-depth discussion. Additionally, Figure 1 graphically illustrates the overall mechanisms of ferroptosis, complementing the tabular information and further facilitating understanding.

**Table 1:** Core mechanisms of ferroptosis.

Core mechanisms	Description	Mode of action	Related signaling pathways	Key molecules or enzymes
iron metabolism imbalance	The intracellular iron ion concentration abnormally increases, leading to an increase in ROS generation.	Catalyzes the production of OḤ free radicals from H2O2 through the Fenton reaction.	Hepcidin-HFE-Transferrin Axis	DMT1, TfR, FPN, Hepcidin, Hfe, Tf, Hepcidin
Lipid peroxidation	PUFAs are attacked by ROS to form lipid peroxides, damaging the cell-membrane structure.	Inhibits the lipid peroxidation process and reduces LOOH accumulation.	SLC7A11/GPX4 Pathway, FSP1-CoQ10 System	GPX4, ACSL4, LPCAT3, FSP1, CoQ10
Antioxidant system failure	The antioxidant defense ability regulated by NRF2 decreases, and the GSH level drops.	Activates the NRF2 signaling pathway and up-regulates the expression of antioxidant genes.	NRF2-ARE Pathway, GSH Synthesis Pathway	NRF2, Keap1, NQO1, HO-1, GCLC/GCLM
Mitochondrial dysfunction	The mitochondrial membrane potential is lost, ATP production decreases, and apoptosis signals are activated.	Mitochondrial Outer-Membrane Permeabilization Mediated by the Bcl2 Family, Mitophagy	Bax, Bak, VDAC, Cytochrome c, PINK1, Parkin E3	

### 2.1 Iron metabolism imbalance

Iron exhibits dual physiological-pathological roles in the nervous system. As an essential enzymatic cofactor, it supports oxygen transport, DNA synthesis, and myelin integrity through heme and sphingolipid biosynthesis. Iron homeostasis is maintained through three regulatory tiers: Cellular uptake via transferrin receptor 1 (TfR1)-mediated endocytosis, modulated by the iron-responsive element/iron-regulatory protein (IRE/IRP) system that monitors intracellular iron levels (Zhang et al., 2023; Chen et al., 2022); Intracellular storage within ferritin nanocages that sequester redox-active Fe^2+^, preventing oxidative stress and lipid peroxidation while undergoing IRP-IRE-mediated translational control. Systemic regulation via the hepcidin-ferroportin (FPN) axis, where hepatic hepcidin degrades FPN during overload to limit plasma iron efflux, while macrophage FPN mediates iron recycling into transferrin. Dysregulation initiates a pathological cascade: excess Fe^2+^ drives Fenton reaction-generated hydroxyl radicals (·OH), triggering lipid peroxidation cascades that culminate in membrane disintegration and ferroptosis. This coordinated regulatory network underscores the critical balance required to maintain iron’s beneficial roles while preventing its neurotoxic potential.

### 2.2 Lipid peroxidation

Lipid peroxidation is the oxidative process involving polyunsaturated fatty acids mediated by reactive oxygen species or reactive nitrogen species and constitutes a fundamental mechanism of ferroptosis (Dixon et al., 2012). Lipid peroxidation is divided into three stages—initiation, propagation, and termination—mediated by free radicals (Xu et al., 2024). In the initiation stage, reactive oxygen species (ROS; such as hydroxyl radicals) are generated through the Fenton reaction and attack polyunsaturated fatty acid (PUFA; Kwun and Lee, 2023); in the propagation stage, lipid radicals combine with oxygen molecules to generate lipid peroxyl radicals, which further target other PUFA molecules, leading to the formation of lipid hydroperoxide (LOOH) and initiating a cascade of reactions (Xu et al., 2024); in the termination stage, the antioxidant system, including GPX4, reduces lipid peroxides to non-toxic lipid alcohols (LOH), interrupting the chain reaction (Wei et al., 2023). Lipid peroxidation predominantly creates three harmful byproducts, which are highly cytotoxic, capable of damaging the integrity of cell membranes, increasing membrane permeability, leading to ionic homeostasis imbalance, mitochondrial dysfunction, and endoplasmic reticulum stress, finally leading to cell death (Kwun and Lee, 2023).

### 2.3 Failure of antioxidant system

Nuclear factor erythroid 2-related factor 2 (NRF2) orchestrates cellular antioxidant defense by directly regulating glutathione (GSH) biosynthesis and GPX4 expression through transcriptional control of Glutamate-Cysteine Ligase Catalytic Subunit(GCLC)/ Glutamate-Cysteine Ligase Modifier Subunit(GCLM) and GPX4 (Fan et al., 2024). This redox guardian operates via three synergistic mechanisms: primarily by enhancing GSH synthesis under oxidative stress, secondly through maintaining GSH availability to preserve GPX4 activity (Chen et al., 2022), and finally via ferritin stabilization to suppress iron-driven lipid peroxidation. The NRF2-GPX4 axis specifically neutralizes LOOH into non-toxic lipid alcohols, thereby blocking membrane destabilization cascades (Wei et al., 2023).

Under pathological conditions, systemic collapse of this defense network occurs through multiple interconnected mechanisms: epigenetic silencing (e.g., GPX4 promoter hypermethylation) impairs transcriptional activation (Wei et al., 2020), ROS overconsumption coupled with Nicotinamide Adenine Dinucleotide Phosphate (NADPH) deficiency and cysteine transport defects deplete GSH reserves, while electrophilic metabolites inactivate GPX4 via post-translational modification (Yang et al., 2022). Ultimately, the failure of these antioxidant systems leads to uncontrolled lipid peroxidation and iron overload, culminating in ferroptosis.

### 2.4 Mitochondrial dysfunction

Mitochondrial dysfunction exacerbates ferroptosis through iron-ROS crosstalk (Yan et al., 2024). Iron accumulation damages respiratory chain complexes (I/III), reducing ATP synthesis while increasing ROS (Javadov, 2022). Released ROS further oxidize mitochondrial lipids and mitochondrial DNA (mtDNA), perpetuating dysfunction. NCOA4-mediated ferritinophagy releases Fe^2+^ from ferritin stores, while mitochondrial iron export via divalent metal transporter 1(DMT1) enriches the labile iron pool (LIP; Barra et al., 2024). This Fe^2+^ fuels Fenton reactions, generating hydroxyl radicals that initiate acyl-CoA synthetaselong-chain family member 4 (ACSL4) / lysophosphatidylcholine acyltransferase 3(LPCAT3)-dependent lipid peroxidation: ACSL4 activates polyunsaturated fatty acids (PUFAs, e.g., arachidonic acid, AA/ adrenic acid, AdA), and then LPCAT3 incorporates them into membrane phospholipids (phosphatidylethanolamine-arachidonic acid/ adrenic acid, PE-AA/AdA), thus making them susceptible to oxidation by lipoxygenases (ALOXs; Feng et al., 2022). Mitophagy’s dual role depends on stress severity: pathological mitophagy (e.g., via PINK1/Parkin) releases iron and promotes ferroptosis, whereas basal mitophagy maintains mitochondrial quality to suppress ROS (Chang et al., 2024).

## 3 Ferroptosis and neurological diseases

### 3.1 Ferroptosis and neurodegenerative diseases

Neurodegenerative diseases pose a severe threat to human health, with the ferroptosis mechanism playing a central and indispensable role in the pathogenesis of AD and PD. These diseases commonly feature selective neuron degeneration and functional loss, and the mechanism of ferroptosis is intricately linked to these pathogenic events.

A gradual decline in cognitive function characterizes AD. Critically, the excessive buildup of iron within the brain establishes a self-sustaining cycle: iron accumulation promotes *β*-amyloid (Aβ) aggregation and tau hyperphosphorylation (Tran et al., 2022), while Aβ plaques in turn sequester iron via their metal-binding domains, increasing local Fenton reaction activity (Tang et al., 2024). This iron-amyloid synergy generates lipid peroxidation products like 4-HNE, which covalently modify tau proteins at Lys311 residues, accelerating neurofibrillary tangle formation (Zhang et al., 2025). Notably, GPX4 activity in AD hippocampi is reduced to that of age-matched controls, directly linking ferroptosis to synaptic loss.

PD represents a paradigm of ferroptosis-driven neurodegeneration. The substantia nigra’s unique vulnerability stems from its relatively high iron content, which is higher than many other brain regions. This iron content synergizes with dopamine’s auto-oxidative properties to generate lipid peroxides. Mechanistically, mutant leucine-rich repeat kinase 2 (LRRK2) phosphorylates ACSL4 at Ser384, enhancing its enzymatic efficiency and driving PUFA incorporation into nigral neuron membranes (Ding et al., 2023). This LRRK2-ferroptosis interaction creates a pathogenic loop: ferroptosis-induced ROS is known to upregulate LRRK2 kinase activity, and the activated LRRK2 further promotes the phosphorylation of *α*-synuclein at Ser129, which is believed to increase its neurotoxicity (Derry et al., 2020).

Amyotrophic Lateral Sclerosis (ALS) is also closely associated with ferroptosis. Ferroptosis directly leads to the degeneration of motor neurons through GPX4 inactivation and lipid peroxidation and interacts synergistically with genetic factors (such as Superoxide Dismutase 1, SOD1 mutants) and pathological proteins (such as TDP-43 aggregates). SOD1 mutants activate ferroptosis by disrupting metal ion homeostasis, while ferroptosis induces mitochondrial dysfunction, further exacerbating oxidative stress and energy metabolism collapse (Wang L. Q. et al., 2024; Esposto and Martic, 2021). Although the role of ferroptosis in ALS has been recognized to some extent, its specific mechanisms still require further research. Future studies need to investigate the differential roles of ferroptosis in different ALS subtypes and develop more effective therapeutic targets.

Huntington’s disease (HD) is an autosomal dominant neurodegenerative disorder caused by mutant huntingtin (mHTT; Yao et al., 2024). mHTT disrupts mitochondrial function, leading to iron (Fe^2+^) accumulation in striatal neurons, Fenton reaction-induced ROS, and lipid peroxidation. It also impairs GSH synthesis, reducing GPX4 and xCT (SLC7A11) activity, exacerbating oxidative damage. In summary, these cascading events driven by mHTT-induced mitochondrial dysfunction highlight the intricate pathogenesis of HD and offer potential therapeutic targets for intervention.

The common mechanisms of ferroptosis in neurodegenerative diseases provide important directions for exploring broad-spectrum therapeutic targets. In the future, intervention strategies targeting ferroptosis, such as iron chelators, antioxidants, and GPX4 activators, are anticipated to yield novel advancements in the management of these ailments.

### 3.2 Ferroptosis and acute brain injury

Acute brain injury (ABI), including stroke and Traumatic Brain Injury (TBI), is a significant contributor to elevated morbidity and mortality rates. Research indicates that ferroptosis significantly contributes to the pathogenic mechanisms of acute brain damage. After acute brain injury, the activation of oxidative stress and inflammatory reactions results in the buildup of intracellular iron, subsequently triggering ferroptosis (Shen et al., 2020). In acute brain injuries, ferroptosis exacerbates neuronal death through iron metabolism disorders and lipid peroxidation. In TBI, the disruption of the blood–brain barrier enables iron ions to penetrate brain tissue. There, ROS produced during the Fenton reaction trigger lipid peroxidation and undermine the integrity of the cell membrane. Simultaneously, the body compensatorily upregulates TF to uptake iron, forming a vicious cycle. In ischemic stroke, ischemia–reperfusion damage is a significant process contributing to neuronal death. During the ischemic phase, iron metabolism imbalance (upregulation of Hmox1 and TF, downregulation of iron transport protein FPN) leads to iron accumulation, while the reperfusion phase accelerates oxidative stress through ROS explosion, synergistically driving ferroptosis mediated by ACSL4-induced lipid peroxidation (Guo et al., 2023). Both phases are accompanied by the secretion of pro-inflammatory cytokines (such as IL-6 and TNF-*α*), activating neuroglial inflammatory responses and further expanding the damage.

Ferroptosis is integral to the pathophysiological mechanisms of SCI. Studies have shown that primary damage caused by mechanical trauma disrupts the integrity of the blood-spinal cord barrier, leading to iron homeostasis imbalance. Excess iron ions produce significant quantities of reactive oxygen species via the Fenton reaction, initiating a cascade of lipid peroxidation. Additionally, SCI also leads to impaired cystine-glutamate antiporter (system Xc-), affecting cysteine uptake and reducing glutathione synthesis, ultimately leading to decreased GPX4 activity and weakening the cell’s antioxidant defense capacity. These factors collectively contribute to a ferroptosis cascade characterized by iron metabolism imbalance, oxidative stress, and lipid peroxidation, Further intensifying neuronal and oligodendrocyte mortality while eliciting inflammatory responses via the release of Damage-Associated Molecular Patterns (DAMPs), forming a vicious cycle that ultimately leads to irreversible damage to spinal cord tissue. Therefore, therapeutic approaches aimed at ferroptosis, including iron chelators, GPX4 activators, and lipid peroxidation inhibitors, may offer novel insights into the management of SCI.

### 3.3 Ferroptosis and neuroimmunological diseases

Neuroimmunological diseases are neurological disorders attributed to irregularities in the immunological system. Ferroptosis drives disease progression in neuroimmunological diseases (such as multiple sclerosis, neuromyelitis optica spectrum disorder, and systemic lupus erythematosus) through dual mechanisms. On the one hand, cell type-specific susceptibility manifests in multiple sclerosis (MS), where oligodendrocytes undergo ferroptosis via the IFN-*γ*-JAK1/STAT1 axis, reducing SLC7A11 expression that impairs cystine import for glutathione synthesis and leading to myelin basic protein (MBP) peroxidation as cell antioxidant capacity weakens due to decreased glutathione levels, the IFN-γ-JAK1/STAT1 axis activation starts a cascade: IFN-γ binds to receptors, phosphorylating STAT1 through JAK1 activation, which then translocates to the nucleus to regulate ferroptosis-related genes (Lin and Lin, 2010). On the other hand, systemic immune dysregulation is exemplified in systemic lupus erythematosus (SLE), where neutrophil ferroptosis synergizes with CaMKIV-CREMα pathway-mediated GPX4 suppression to potentiate NETosis (Li et al., 2021). These ferroptosis-related mechanisms thus hold promise for developing new therapies for neuroimmunological diseases.

### 3.4 Ferroptosis and other neurological diseases

Besides the aforementioned ailments, ferroptosis is also intricately linked to numerous other neurological disorders. For instance, the pathogenesis of depression, anxiety, and epilepsy has also been found to involve ferroptosis. In depression, abnormal iron deposition in the prefrontal cortex and hippocampus triggers the accumulation of lipid peroxides through the Fenton reaction, directly damaging synaptic plasticity-related proteins (such as BDNF/TrkB signaling pathway), resulting in atypical neural network connections, a phenomenon closely linked to cognitive deterioration and emotional disturbances in patients (Liu et al., 2024); animal experiments further confirm that GPX4 activity is significantly reduced in the brains of chronic stress model mice (Zhang et al., 2024). The pathological process of epilepsy is also deeply intertwined with ferroptosis; iron overload in epileptic foci promotes the generation of lipid peroxides through ACSL4-mediated arachidonic acid metabolism, while decreased GPX4 activity further weakens antioxidant defenses, forming a vicious cycle of “neuronal hyperexcitability-oxidative stress.” Preclinical studies show that using Liproxstatin-1 to inhibit ferroptosis can significantly reduce the frequency of seizures and protect hippocampal neurons (Batista et al., 2021). Activation of the HIF-1α/H -1 pathway is evident as hippocampal neurons in epileptic animal models and epilepsy patients’ brain tissues show marked HIF-1α and HO-1 upregulation, positively correlating with ferroptosis markers like lipid peroxides, ACSL4 increase, and GPX4 inactivation (Liang Z. et al., 2023). Emerging clinical evidence from patients with GABAB receptor encephalitis-associated epilepsy reveals significant elevation of cerebrospinal fluid ferroptosis biomarkers such as malondialdehyde (MDA) and 4-HNE, along with glutathione depletion and ACSL4 upregulation, mechanistically linked to exosomal miR-92a-3p-mediated NF2/P-YAP pathway suppression that worsens neuronal iron overload and lipid peroxidation (Zhang et al., 2025). In vascular dementia (VD), chronic cerebral ischemia drives ferroptosis through dual mechanisms—iron metabolism imbalance (upregulation of transferrin and its receptor TfR1 and downregulation of iron export protein FPN) results in the buildup of free iron within neurons, while oxidative stress accelerates lipid peroxidation by inhibiting GPX4 synthesis and upregulating ACSL4. Clinical data indicate that concentrations of indicators such as MDA and 4-HNE in the cerebrospinal fluid of VD patients are negatively correlated with cognitive function, while the iron chelator Deferiprone can delay disease progression by restoring iron homeostasis (Yan and Zhang, 2019; Fu et al., 2023). These findings show that ferroptosis is integral to neuropsychiatric disorders and set the stage for developing treatments that can address multiple diseases.

**Figure 2:**
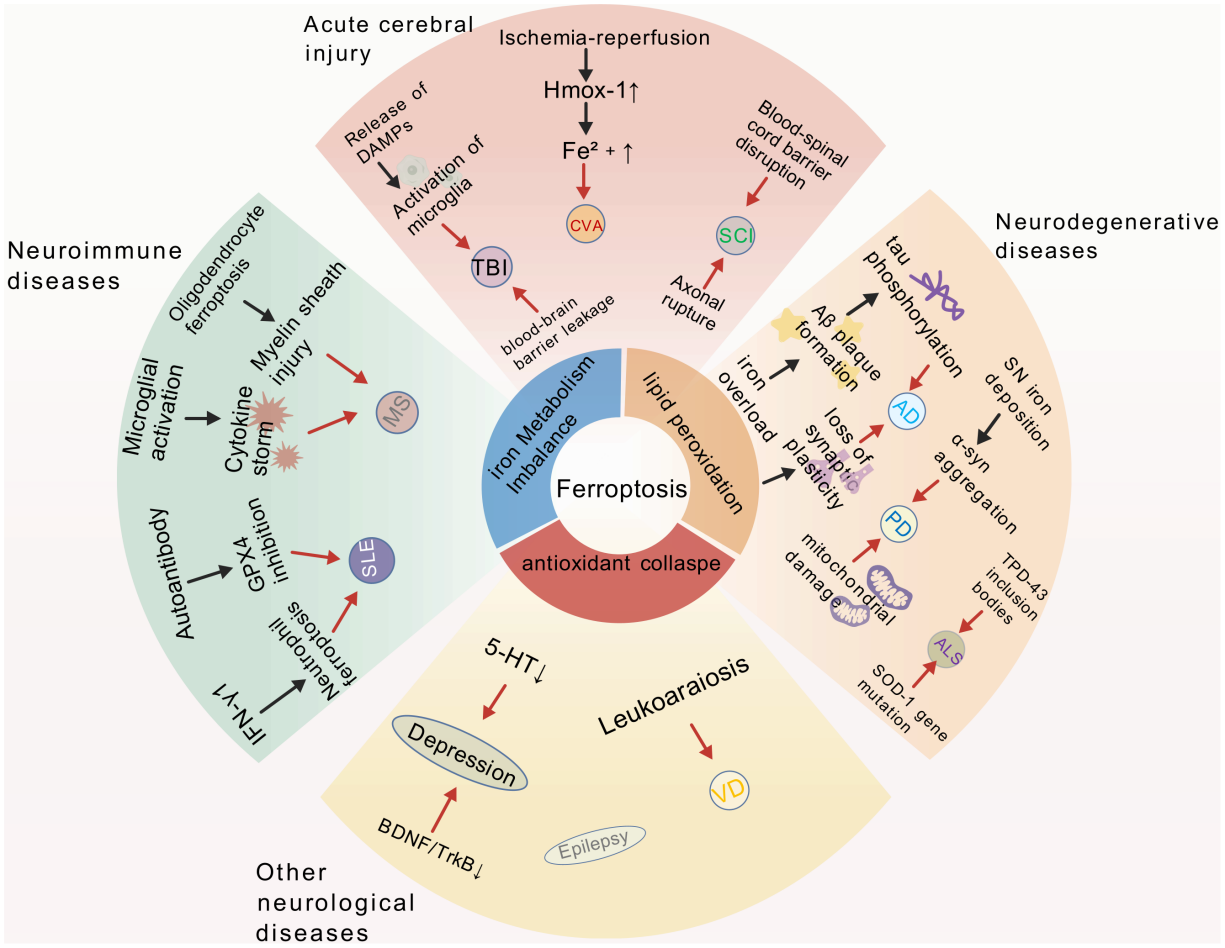
Created with Biogdp.com (Jiang et al., 2025). This diagram illustrates the associations between ferroptosis and various nervous system diseases. In the central part of the diagram, ferroptosis is labeled. The surrounding ring points out the key factors related to ferroptosis. Different sectors radiating out from the center correspond to various types of nervous system diseases. Overall, it elucidates the function and associated mechanisms of ferroptosis in the onset and progression of various neurological disorders.

Table 2 provides a comprehensive summary of ferroptosis in relation to various neurological diseases covered in this section, including neurodegenerative, acute brain injury, neuroimmunological, and other neurological conditions. Additionally, Figure 2 graphically depicts key aspects of these relationships, offering a visual complement to the tabular data.

**Table 2:** Related mechanisms between ferroptosis and nervous system diseases.

Disease type	Representative diseases	Ferroptosis-related mechanisms	Key regulatory molecules
Neurodegenerative diseases	AD	Hippocampal iron deposition, lipid peroxidation, tau protein phosphorylation	Tf, FPN, GPX4, APP, Tau
	PD	Iron overload in substantia nigra, α-syn aggregation-induced oxidative stress, mitochondrial dysfunction	α-syn, LRRK2, NCOA4, FPN
	ALS	Lipid peroxidation, motor neuron degeneration, SOD1 mutation leading to mitochondrial iron accumulation, TDP-43 inclusion-related oxidative damage	HMGB1, IL-6, HO-1, TfR1
	HD	mHTT-induced mitochondrial iron overload, GSH depletion in striatal neurons	mHTT, GPX4, xCT, Nrf2
Acute neurological injury diseases	TBI	Blood–brain barrier disruption, iron ion infiltration, DAMPs activating microglia	FPN, GPX4, IL-6, TNF-α
	CVA	Ischemia-reperfusion inducing Hmox1↑, free iron release, ACSL4—mediated lipid peroxidation	Hmox1, NF-κB, ACSL4, GPX4
	SCI	Iron accumulation, myelin sheath and axon damage, mitochondrial dysfunction	SLC7A11, SLC3A2↓, GPX4
Neuroimmune diseases	MS	Ferroptosis of oligodendrocytes leading to myelin sheath damage, microglia activation	MBP, CD68
	SLE	Chronic cerebral ischemia, imbalance of iron uptake and excretion	SLC7A11, GSH, SLC7A11, GPX4
Other nervous system diseases	Depression	Prefrontal iron deposition, BDNF/TrkB signaling inhibition exacerbating oxidative damage	BDNF, TrkB, FTL, 5-HT
	Epilepsy	ACSL4-mediated hippocampal lipid peroxidation, decreased GPX4 activity leading to abnormal neuronal firing	ACSL4, GPX4, GABA, NMDA
	VD	Chronic ischemia induces FPN down-regulation, causing cortical iron accumulation and oxidative stress, leading to leukoaraiosis	FPN, HIF-1α, VEGF, HO-1

Through an in-depth exploration of neurological diseases, we clearly recognize that ferroptosis is crucial in the onset and progression of neurological diseases. Ferroptosis disrupts iron metabolism, promotes lipid peroxidation, and triggers inflammatory-immune imbalances, all of which are key factors in disease progression. This leads to irreversible neuronal damage and significantly diminishes patients’ quality of life. As our understanding of ferroptosis improves, we will explore therapeutic drugs and intervention strategies that may reverse disease progression, enhance prognosis, and improve the quality of life, thus revitalizing treatment approaches for neurological diseases.

## 4 Interventions targeting ferroptosis related to neurological diseases

**Figure 3:**
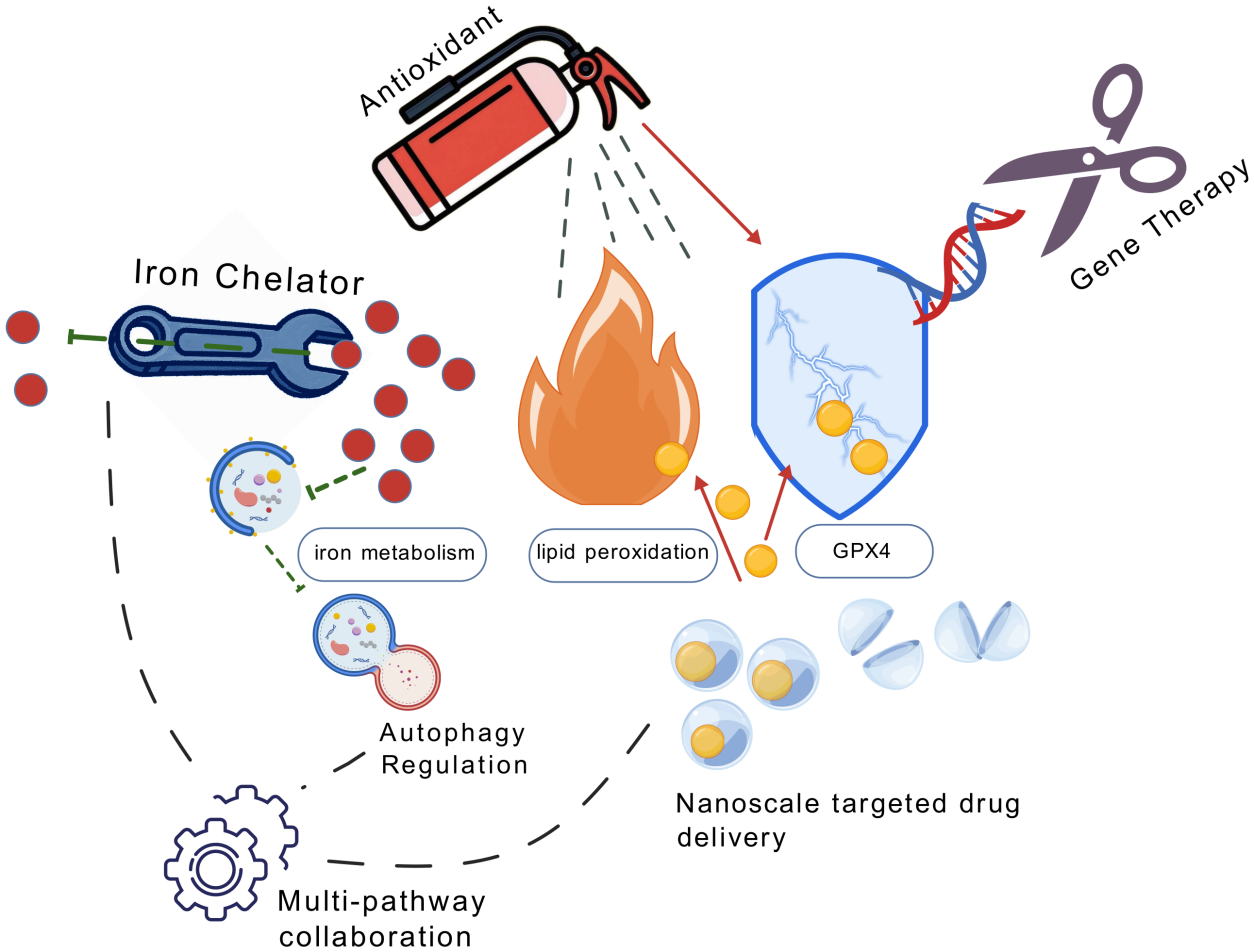
Created with BioGDP (Jiang et al., 2025). This schematic diagram centers on the three core elements of ferroptosis (iron metabolism imbalance, lipid peroxidation, antioxidant collapse), with six categories of targeted therapeutic strategies surrounding it. Through iron chelators, antioxidants, nano-drug delivery, gene regulation, autophagy induction, and multi-pathway coordination, dynamic arrows visually display the action pathways of each therapy—from removing excessive iron ions and inhibiting the lipid oxidation storm to repairing the antioxidant barrier. Finally, cross-mechanism coordinated intervention is achieved through the interlocking of gears.

Ferroptosis, an iron-dependent cell death modality instigated by lipid peroxidation and inflammation, is implicated in neurodegenerative disorders and cerebral injuries. The underlying mechanisms are being elucidated: iron dyshomeostasis potentiates oxidative stress, and antioxidant system impairment exacerbates membrane vulnerability. Owing to the intimate connection between ferroptosis and intracellular iron accretion as well as lipid peroxidation, identifying novel targets for ferroptosis inhibitors represents a crucial avenue for future research. Pharmacotherapies targeting these pathways may offer substantial therapeutic value. Table 3 presents a detailed overview of the relationships between ferroptosis and nervous system diseases, listing disease types, representative diseases, ferroptosis-related mechanisms, and key regulatory molecules. Meanwhile, Figure 3 graphically demonstrates various therapeutic approaches targeting ferroptosis-related processes in the nervous system.

**Table 3:** Drugs/molecules targeting ferroptosis in neurological disorders.

Drug/molecule	Mechanism of action	Neurological disorder & effect	Reference
Ferrostatin-1 (Fer-1)	Inhibits lipid peroxidation and ROS production by scavenging lipid radicals.	IS /TBI: Reduces neuronal death and edema.	You et al. (2024)
Deferoxamine (DFO)	Iron chelator reduces intracellular iron overload.	AD: Mitigates iron-driven amyloid-beta toxicity.	Entezari et al. (2022); Marupudi and Xiong (2024)
Clioquinol (CQ)	Reduces cerebral iron content and ROS; crosses the blood–brain barrier.	PD: Attenuates dopaminergic neuron loss.	Teil et al. (2022)
Lactoferrin (Ltf)	Binds free iron; inhibits iron-dependent lipid peroxidation	AD/PD Models: Reduces neuroinflammation and oxidative damage	Yong et al. (2023)
Resveratrol NPs (Res-NPs)	Scavenges ROS; enhances mitochondrial function.	AD: Improves cognitive deficits and reduces amyloid plaque burden.	Pasinetti et al. (2015)
Dauricine	Upregulates GPX4; suppresses lipid peroxidation.	PD: Protects dopaminergic neurons from ferroptosis.	Peng et al. (2022)
Curcumin NPs (Cur-NPs)	Boosts GPX4 activity; inhibits ACSL4-mediated lipid peroxidation.	AD/Stroke: Reduces neuronal death and improves memory.	Panzarini et al. (2020)
N-Acetylcysteine (NAC)	Inhibits ALOX5-mediated toxic PUFA oxidation; replenishes glutathione.	ALS: Slows motor neuron degeneration.	Raghu et al. (2021)
Isorhynchophylline (IRN)	Activates SLC7A11 via miR-122-5p/TP53 pathway.	ICH: Reduces ferroptosis in peri-hematomal neurons.	Deng et al. (2021)
Baicalin	Upregulates GPX4 and SLC7A11; stabilizes redox balance.	HD: Suppresses striatal neuron loss.	Zhou et al. (2019)
Crocin	Activates Nrf2 nuclear translocation; enhances GPX4/SLC7A11 expression	AD/PD: Counters amyloid-beta and *α*-synuclein-induced ferroptosis.	Yousefsani et al. (2021)
Pioglitazone	PPARγ agonist; synergizes with Nrf2/ARE-GPX4 pathway.	Stroke/AD: Enhances antioxidant defenses and reduces neuronal damage.	Feng (2023)

### 4.1 Iron chelation therapy

Iron chelators are effective in treating iron metabolism imbalances in neurological diseases by removing free iron and inhibiting ferroptotic cell death and oxidative stress. Iron chelators like Deferoxamine (DFO) and Deferasirox are utilized for specific therapeutic purposes in neurological disorders. Research has demonstrated that DFO can effectively decrease cerebral iron concentrations, alleviate oxidative stress damage, and improve symptoms of neurodegenerative diseases (Marupudi and Xiong, 2024). In TBI, DFO improves neurological function by lowering levels of free iron in the brain and inhibiting ferroptosis markers (ACSF2, PTGS2) and lipid peroxidation products (4-HNE, COX2; Cheng et al., 2023). In SCI, DFO upregulates hypoxia-inducible factor-1α (HIF-1α) and vascular endothelial growth factor, facilitating angiogenesis and tissue regeneration (Tang G. et al., 2020). A randomized trial has confirmed that DFO can alleviate iron overload after spinal cord injury and improve the inflammatory microenvironment following spinal cord injury by promoting phagocytosis and removal of myelin debris by microvascular endothelial cells (MECs; Yao et al., 2019). For PD, multifunctional nanoregulators based on DFO can specifically target dopaminergic neurons, selectively removing mitochondrial iron accumulation and reactive oxygen species, restoring iron homeostasis, and alleviating motor dysfunction (Cerasuolo et al., 2023).

In oral iron chelation therapy, in Friedreich’s ataxia (FRDA), Deferasirox (20 mg/kg/day) significantly reduces iron deposition in the cerebellum and spinal cord, decreases lipid peroxidation markers (MDA), and slows the progression of ataxia symptoms (Starr et al., 2021). For AD, research by Ping Kwan et al. found that in AD and tau protein deposition models, it can reduce the formation of neurofibrillary tangles (NFT) in cerebral tissue and decrease amounts of phosphorylated tau protein, thereby exerting protective effects. At the same time, it reinstates the expression of proteins associated with synaptic plasticity, including postsynaptic density protein 95 (PSD-95). It improves spatial memory ability in model mice (Kwan et al., 2022). Prior studies have experimentally confirmed that Deferasirox can significantly reduce iron content in the brain tissue of APP/PS1 HAMP−/− mice, alleviate mitochondrial morphological damage, and improve learning and memory ability (Zou et al., 2019). The development of new iron chelators is also underway, such as an oral iron chelator FBS0701, which reduces striatal neuronal death in HD models by blocking the formation of iron-dopamine complexes. In terms of clinical translation, a Phase II trial (NCT04510719) showed that Deferasirox combined with vitamin E can delay brain atrophy in multiple sclerosis (MS) patients by inhibiting oligodendrocyte ferroptosis. Collectively, these studies indicate that the core mechanisms of Deferasirox include targeting the clearance of pathological iron accumulation, inhibiting the Fenton reaction, and regulating key pathways of ferroptosis, providing a treatment strategy that combines safety and accessibility for neurodegenerative diseases and acquired nerve injuries.

### 4.2 Antioxidants

Antioxidants have become an essential strategy for treating neurological diseases by inhibiting lipid peroxidation and blocking the ferroptosis cascade. Lipophilic free radical scavengers (RTAs) like Ferrostatin-1 and Liproxstatin-1 have protective benefits by neutralizing lipid peroxyl radicals (Tang S. et al., 2020). In TBI, Ferrostatin-1 diminishes cerebral iron accumulation and neuronal apoptosis, enhancing cognitive performance; in ischemic stroke models, intranasal administration of Liproxstatin-1 can diminish the amount of cerebral infarction and mitigate motor impairments (Shen et al., 2020). Fatty acid modification strategies are also effective; deuterated polyunsaturated fatty acids (D-PUFAs) inhibit lipid peroxidation chain reactions by stabilizing the bis-allylic structure, while Omega-3 fatty acids augment neuronal antioxidant capacity by elevating the ratio of monounsaturated fatty acids in membrane phospholipids.

Targeting the antioxidant defense system is another core direction. The FSP1/CoQ10 system neutralizes lipid free radicals through reduced CoQ10; in subarachnoid hemorrhage (SAH) models, liposome-encapsulated CoQ10 can activate FSP1 and inhibit neuronal ferroptosis (Peng et al., 2024; Wang et al., 2023). Mitochondrial-targeted antioxidants such as MitoTEMPO precisely clear mitochondrial ROS, alleviating dopaminergic neuronal degeneration in PD models (Chen et al., 2023). Additionally, natural antioxidants, including vitamin E and N-acetylcysteine (NAC), exhibit neuroprotective effects in neurological diseases by replenishing glutathione or directly neutralizing ROS. New combination therapies are being explored; for example, when Deferasirox is used in conjunction with other antioxidants (such as ebselen), it may enhance its neuroprotective effects. In patients with Friedreich’s ataxia, the combination of Deferoxamine and ebselen significantly improves neurological function (Tian et al., 2022). Edaravone combined with Liproxstatin-1 can synergistically inhibit oxidative damage and ferroptosis in ALS models. These multi-pathway intervention strategies provide a precise therapy basis for neurodegenerative disorders and acute brain injuries.

### 4.3 Multi-pathway coordinated regulation of ferroptosis

Multi-target strategies for ferroptosis protect nerves by strengthening endogenous defenses, mending antioxidant systems, and halting oxidative damage. Activating cellular protective factors is crucial. The NRF2 pathway neutralizes lipid peroxides via antioxidant enzymes like HO-1. Sulforaphane helps in Alzheimer’s (reducing A*β*, enhancing cognition; Hua et al., 2022), and dimethyl fumarate fights multiple sclerosis by blocking oligodendrocyte ferroptosis through Nrf2 (Hammer et al., 2018). Anti-inflammatory cytokines IL-10 and TGF-β show promise in Parkinson’s and stroke by curbing neuroinflammation. Targeting GPX4 restores antioxidant enzymes; selenium aids Alzheimer’s by boosting GPX4 (Liang X. et al., 2023), and sulfanilamide increases antioxidant reserves (Sacramento et al., 2022). ACSL4 inhibitors like rosiglitazone reduce membrane sensitivity and infarct size. Lipophilic antioxidants and iron chelators work together to block radicals and cut oxidative damage (Aydemir et al., 2020). These strategies can form a treatment plan for neurodegenerative diseases and brain injuries, and future work should focus on optimizing delivery and translation for better results.

### 4.4 Other treatments

#### 4.4.1 Gene therapy

Gene therapy offers a new direction for neurological diseases by precisely regulating genes related to ferroptosis. For example, enhancing GPX4 gene expression using CRISPR/Cas9 technology can improve the antioxidant capacity of neurons and directly inhibit lipid peroxidation (Lam et al., 2024); delivering the iron transport protein (FPN1) or ferritin heavy chain (FTH1) genes via AAV vectors can promote iron export or storage, reducing free iron accumulation (Prasad and Hung, 2021). Additionally, delivering the Nrf2 gene can activate endogenous antioxidant pathways to combat ferroptosis. Despite its broad prospects, optimizing delivery systems (such as selecting AAV serotypes) to improve targeting and addressing safety issues in clinical translation remains necessary. In the future, combining gene therapy with iron chelators and other strategies may achieve more effective neuroprotection.

Base editing, a CRISPR-based technology, combines catalytically impaired Cas9 with deaminase enzymes to directly convert nucleotides without double-strand DNA breaks, offering new treatment options for genetic diseases. In FRDA, caused by a GAA trinucleotide repeat expansion in the FXN gene, adenine base editors (ABEs) could potentially treat the disease by converting A-T to G-C base pairs within the expanded repeats in the FXN gene. This might increase frataxin production and relieve FRDA symptoms (Derry et al., 2020). However, base editing for FRDA treatment has off-target issues and requires future improvements.

#### 4.4.2 Precise regulation of autophagy-iron metabolism homeostasis

Autophagy regulation provides a new direction for balancing iron metabolism. Autophagy drives ferroptosis by degrading ferritin and releasing free iron. In Parkinson’s disease models, chloroquine (CQ) reduces ferritin degradation by inhibiting lysosomal acidification, lowering levels of free iron in the brain while restoring mitochondrial function (Peng et al., 2023); in ischemic stroke models, low-dose bafilomycin A1 (BafA1) targets and blocks NCOA4-mediated ferritinophagy, reducing cerebral infarction volume (Wang X. et al., 2024).

#### 4.4.3 Nanotargeted drug delivery enhancement platform

Nano-drug delivery systems significantly enhance the precision of ferroptosis interventions through targeted delivery and intelligent drug release technologies. Transferrin receptor-targeted liposomes carrying Ferrostatin-1 can mediate transport across the blood–brain barrier via TfR on the surface of brain capillary endothelial cells, reducing the accumulation of lipid peroxides; exosome-Liproxstatin-1 complexes utilize the natural targeting ability of bone marrow mesenchymal stem cell exosomes, preferentially enriching around Aβ plaques in AD models, increasing the survival rate of hippocampal neurons. By employing multidimensional coordinated interventions, these emerging strategies are paving new pathways for treating complex neurological diseases.

#### 4.4.4 RNA interference technology

This approach employs siRNA or shRNA to silence pro-ferroptotic genes. Targeting ACSL4, a key lipid metabolism enzyme, reduces neuronal MDA levels by 54% (Liao et al., 2024). Delivery of anti-ferroptotic miRNAs, such as miR-137-3p, has emerged as a promising approach for modulating ferroptosis-related processes. miR-137-3p can bind to the 3’untranslated region (3’UTR) of SLC7A11 mRNA. By targeting SLC7A11 mRNA, miR-137-3p can enhance cystine uptake and increase glutathione synthesis (Xu et al., 2023; Mahmoudi-Lamouki et al., 2023). Novel biodegradable lipid nanoparticles (LNPs) exhibit 22% blood–brain barrier penetration efficiency but require repeated administration every 3 weeks (Shankar et al., 2018). As an emerging technology, RNA interference still requires further research. It holds potential to become a therapeutic approach for neurological diseases caused by ferroptosis.

## 5 Conclusion and perspectives

Ferroptosis is intricately linked to neurological disorders. The main mechanisms include imbalances in iron metabolism, lipid peroxidation, antioxidant system failure, and mitochondrial dysfunction, all of which interweave to promote the ferroptosis process. Currently, interventions targeting ferroptosis encompass multiple aspects, with transferrin, hepcidin, and others participating in regulating iron metabolism; iron chelators can improve iron metabolism disorders; various antioxidants, enzymes, and related regulatory substances can alleviate oxidative stress and lipid peroxidation; gene therapy can precisely regulate gene expression, and traditional Chinese medicine can intervene through multiple components and compound multi-target approaches, but all face different challenges. In summary, research on the mechanisms of ferroptosis brings new directions for treating neurological diseases. However, translating these findings into clinically effective applications necessitates deeper exploration of molecular pathways, refinement of therapeutic strategies, and resolution of existing technical challenges. In this context, ferroptosis biomarkers such as the ferritin/ GSH ratio in cerebrospinal fluid (CSF) exhibit significant diagnostic potential.

Future focus should be on two directions: on one hand, deepening basic research, including more detailed exploration of ferroptosis signaling pathways, clarifying the mechanisms of action of various molecules and the cell-type-specific response processes; optimizing animal models to more accurately reflect disease pathology, using advanced imaging techniques to observe the ferroptosis process dynamically; actively seeking new molecular markers of ferroptosis and studying their value in disease diagnosis, progression monitoring, and treatment efficacy assessment. Moreover, the integrated application of metabolomics and single-cell sequencing provides a powerful strategy to investigate spatiotemporal heterogeneity of ferroptosis in neurological disorders. Metabolomics comprehensively profiles ferroptosis-associated metabolic dynamics, while single-cell sequencing resolves cell-type-specific susceptibility through transcriptional/epigenetic characterization of neural subpopulations, enabling precise mapping of ferroptotic vulnerability across distinct brain regions. On the other hand, we should advance the development of clinical applications. Firstly, develop particular regulatory drugs; secondly, construct intelligent drug delivery systems. Moreover, dynamically assess the ferroptosis activity of patients based on biomarkers such as 4-HNE and free iron in cerebrospinal fluid. By combining single-cell sequencing technology to analyze individual metabolic heterogeneity, we can establish a personalized treatment system and guide the combined drug treatment plan. With the comprehensive application of ferroptosis molecular probes, gene editing tools, and artificial intelligence prediction models, treatment strategies will gradually shift from “generalized inhibition” to “spatiotemporal precise intervention.”

## Glossary

ABI: Acute Brain Injury
AD: Alzheimer’s Disease
ALS: Amyotrophic Lateral Sclerosis
APP: Amyloid Precursor Protein
Aβ: β-Amyloid Protein
DAMPs: Damage-Associated Molecular Patterns
DFO: Deferoxamine
D-PUFA: Deuterated Polyunsaturated Fatty Acids
GCLC: Glutamate-Cysteine Ligase Catalytic Subunit
GCLM: Glutamate-Cysteine Ligase Modifier Subunit
HD: Huntington’s disease
FPN: Ferroportin
Ft: Ferritin
GPX4: Glutathione Peroxidase 4
GSH: Glutathione
4-HNE: 4-Hydroxynonenal
IREs: Iron-Response Elements
IRPs: Iron-Regulatory Proteins
LOOH: Lipid Peroxides
LRRK2: Leucine-Rich Repeat Kinase 2
MDA: Malondialdehyde
MECs: Microvascular Endothelial Cells
mtDNA: mitochondrial DNA
mHTT: mutant huntingtin
MS: Multiple Sclerosis
NAC: N-Acetylcysteine
NADPH: Nicotinamide Adenine Dinucleotide Phosphate
NCOA4: Nuclear Receptor Coactivator 4
NFT: Neurofibrillary Tangles
NRF2: Nuclear Factor Erythroid 2-Related Factor 2
PD: Parkinson’s Disease
PUFA: Polyunsaturated Fatty Acid
ROS: Reactive Oxygen Species
RTAs: Radical Scavengers
SAH: Subarachnoid Hemorrhage
SCI: Spinal Cord Injury
System XC: cystine-glutamate antiporter
SLE: Systemic Lupus Erythematosus
SOD1: Superoxide Dismutase 1
TBI: Traumatic Brain Injury
TF: Transferrin
TfR: Transferrin Receptor
VD: Vascular Dementia
